# Collision Tumor of the Ovary: Adult Granulosa Cell Tumor and Mesonephric-like Adenocarcinoma

**DOI:** 10.3390/diagnostics14131412

**Published:** 2024-07-02

**Authors:** Yujin Lee, Mohammad Rizwan Alam, Jin-Hwi Kim, Chan Joo Kim, Su Lim Lee, Kwangil Yim

**Affiliations:** 1 Department of Hospital Pathology, St. Vincent’s Hospital, College of Medicine, The Catholic University of Korea, Seoul 06591, Republic of Korea; 2 Department of Hospital Pathology, Uijeongbu St. Mary’s Hospital, College of Medicine, The Catholic University of Korea, Seoul 06591, Republic of Korea; 3 Department of Obstetrics and Gynecology, Uijeongbu St. Mary’s Hospital, College of Medicine, The Catholic University of Korea, Seoul 06591, Republic of Korea; 4 Department of Radiology, Uijeongbu St. Mary’s Hospital, College of Medicine, The Catholic University of Korea, Seoul 06591, Republic of Korea

**Keywords:** collision tumor, adult granulosa cell tumor, mesonephric-like adenocarcinoma, ovarian cancer, magnetic resonance imaging, pathology

## Abstract

Collision tumors of the ovaries are rare, with only a few reports in the literature. Adult granulosa cell tumors are a relatively common primary tumor component of previously reported collision tumors. The combination of serous and mucinous tumors with adult granulosa cell tumors has been reported in several cases. On the other hand, mesonephric-like adenocarcinomas are rare neoplasms that commonly arise in the uterine corpus and ovaries. In this report, we present the case of a collision tumor composed of an adult granulosa cell tumor and mesonephric-like adenocarcinoma of the ovary in a 63-year-old woman. The initial magnetic resonance imaging findings showed a cystic mass with an internal hemorrhage, which suggested an adult granulosa cell tumor, and a solid mass with different enhancements. Microscopically, the tumor had two distinct components: An adult granulosa cell tumor and a mesonephric-like adenocarcinoma. Recognizing collision tumors consisting of slow-growing and aggressive tumors may prove beneficial in future diagnostic and treatment strategies.

**Figure 1:**
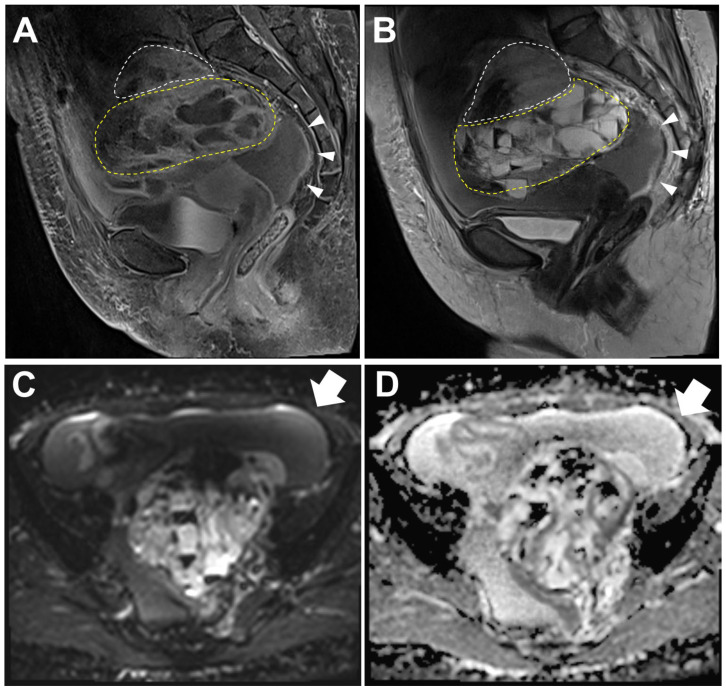
Magnetic resonance imaging (MRI) of the pelvis. Sagittal fat suppression T1-weighted (**A**) and T2-weighted (**B**) images revealed a multiloculated cyst with watery fluid or internal hemorrhage in the left ovary measuring 11.7 cm in size (anterior–posterior extension: 11.1 cm, width: 10.5 cm, oblique craniocaudal extension: 11.7 cm). Based on the multilocular cystic appearance with a macrofollicular pattern filled with watery fluid or hemorrhage, the impression was an adult granulosa cell tumor (AGCT) [1,2,3] and endometrioid carcinoma [4]. A solid component was noted at the cranial side (white dashed line) of the mass, measuring 5.4 cm in size (anterior–posterior extension: 4.0 cm, width: 5.4 cm, oblique craniocaudal extension: 3.5 cm). In this area, fat suppression T1-weighted images exhibited enhancement resembling that of a multilocular cyst (yellow dashed line), whereas the T2-weighted images showed a comparatively lower level of enhancement. In addition, peritoneal thickening was shown (arrowheads). Diffusion-weighted image (**C**) and apparent diffusion coefficient map (**D**) showed ascitic fluid without diffusion restriction (arrow).

Collision tumors of the ovary [5,6,7,8,9,10,11,12,13,14,15,16] are very rare, making their inclusion in differential diagnosis challenging in routine clinical practice. Moreover, ovarian cancers often present with heterogeneous components, so the presence of both solid components and multilocular cysts is not unusual [4,17]. However, in the present case, the enhancement in the solid component in the T2-weighted images was lower than that in the multilocular cystic area. The same part of the tumor usually shows similar enhancement patterns [2,3,4,14,15,16]; therefore, the possibility of a collision tumor was suspected.

A 63-year-old woman visited our outpatient clinic with a left ovarian mass found on an external computed tomography scan. The symptoms at presentation included abdominal discomfort. Preoperative laboratory test results for serum CA125 and CA19-9 levels were within the normal range (10.6 U/mL and 8.73 U/mL, respectively). The patient underwent a Papanicolaou test of routine screening one year ago at an external hospital, which yielded a negative result. An MRI examination was requested for a diagnostic work-up (Figure 1), and a left ovarian mass and a large amount of ascites with mild peritoneal thickening were observed. Ascites may be attributable to a partial mass rupture and hemoperitoneum, and it was necessary to consider this as an early finding indicative of potential peritoneal seeding. The patient underwent a total hysterectomy with bilateral salpingo-oophorectomy, omentectomy, and lymph node dissection. Pathologic examination was performed for the diagnosis.

**Figure 2:**
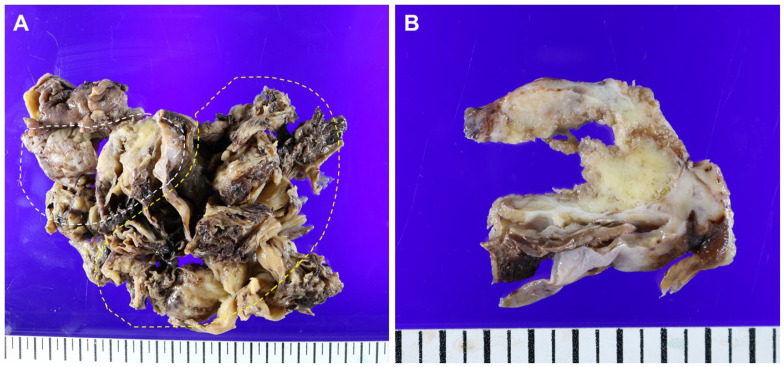
Representative gross findings of the ovary. The left ovary, fragmented, was totally replaced by a tumor obscuring the normal tissue, measuring 18.7 × 15.0 × 3.0 cm (**A**). The tumor involved the ovarian surface with capsular rupture. The mass was heterogeneous and consisted of portions showing multiloculated cysts with internal hemorrhage (yellow dashed line) and a solid area with papillary configuration (white dashed line). The solid area, measuring 5.3 × 4.0 × 3.5 cm, was white-grayish and relatively friable. Due to the fragmentation of the specimen, it was difficult to match the MRI findings exactly, but the solid papillary area (white dashed line) was presumed to be the solid component portion observed on the cranial side (**A**). The cut surface of the solid component was mostly ill-defined, yellow-to-tan, and firm without necrosis (**B**).

Pathological examination revealed a collision tumor with ovarian capsular rupture and uterine serosal implants of a mesonephric-like adenocarcinoma (MLA) component (0.4 × 0.3 cm). The bilateral fallopian tubes and omentum were not involved in the tumor, and there was no lymph node metastasis. The patient was diagnosed with FIGO stage IIA and treated with adjuvant chemotherapy.

**Figure 3:**
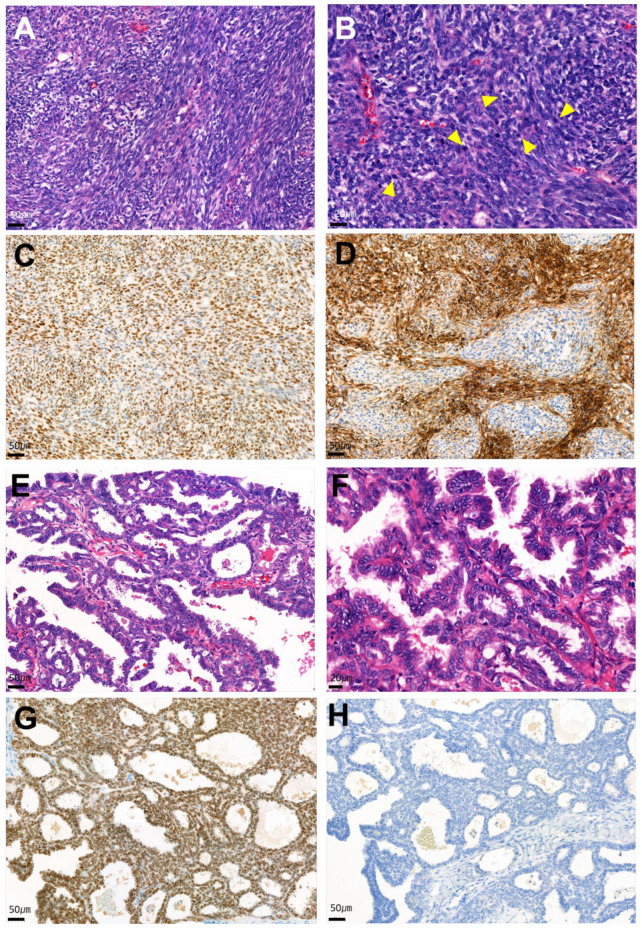
Histological (**A**,**B**,**E**,**F**) and immunohistochemical (**C**,**D**,**G**,**H**) findings of the AGCT (**A**–**D**) and MLA components (**E**–**H**) of the collision tumor. The AGCT cells had oval- to spindle-shaped cells with a fascicular arrangement (×20) (**A**). On high-power magnification (×40), the granulosa cells exhibited pale nuclei with inconspicuous nucleoli and scant cytoplasm. Mitotic figures were frequently seen (yellow arrowheads) (**B**). The tumor cells were positive for WT-1 (×20) (**C**) and CD56a (×20) [18] (**D**) immunostaining. The MLA component of the tumor showed tubuloglandular and papillary growth patterns (×20) (**E**). The nuclei of the tumor cells were vesicular and had grooves with nuclear overlapping, resembling features of papillary thyroid carcinoma on high-power magnification (×40) (**F**). The tumor cells were diffusely positive for GATA3 (×20) (**G**) and negative for TTF-1 (×20) (**H**). In MLA tumors, GATA3 and TTF-1 exhibit either focal or diffuse staining with an inverse pattern, where cells positive for GATA3 are negative for TTF-1 and vice versa [19]. In summary, the immunohistochemistry profile of the AGCT lesion showed positivity for Calretinin, CD99 (diffuse, weak), CD56a (diffuse, strong), and WT-1 (diffuse, strong). The MLA component was positive for CD10 (diffuse, strong), GATA3 (diffuse, strong), PAX-8 (diffuse, strong), and cytokeratin 7 (diffuse, strong). Immunostaining for cytokeratin 20, ER, PR, vimentin, inhibin-alpha, and Napsin-A showed negativity in both components. The detailed information about the antibodies used in this study is summarized in Supplementary Table S1.

Based on these findings, the final diagnosis of a collision tumor with AGCT and MLA components was established. Ovarian collision tumors are rare, with only a few cases reported [5,6,7,8,9,10,11,12,13,14,15,16]. The AGCT is characteristically presented with a solid structure alongside either a unilocular or multilocular cystic morphology, often accompanied by hemorrhagic features within the cyst [20]. These tumors can constitute a significant component of collision tumors that have been documented in prior studies [5,6,9,11,12,13], where they were typically associated with epithelial neoplasms. Several combinations of epithelial and stromal tumors have been reported, and these components can be benign or malignant [21].

On the other hand, MLA arising in the ovary is rare. In human embryogenesis, the mesonephric tubules and ducts are precursors of the male genital tract. In women, rare cases of mesonephric adenocarcinoma arise in the female genital tract from the mesonephric remnants in the vagina and cervix [22]. There have been reports of mesonephric adenocarcinoma of the cervix containing sarcomatous component [23] or mixed with high-grade neuroendocrine carcinoma [24]. A neoplasm in the upper gynecological tract that mimics mesonephric adenocarcinoma is referred to as an MLA because its association with mesonephric remnants has not been demonstrated [25]. MLA are positive for GATA3 [19] and are distinguishable from adenocarcinomas of the upper female genital tract, which morphologically mimic MLA [25]. To date, only two reported cases of collision tumors involving MLA of the ovary have occurred in conjunction with serous tumors [7,26]. These two cases of collision tumors involving MLA of the ovary reported to date have occurred in combination with serous tumors [7,26]. Authors in the literature have proposed that MLA is likely of Müllerian origin, a hypothesis supported by the presence of identical KRAS or NRAS mutations found in mesonephric adenocarcinomas of the uterine cervix or vagina [27,28]. In the present case, KRAS and NRAS molecular tests were performed, and a point mutation in KRAS codon 12 was found.

To the best of our knowledge, the present case is exceptionally unique as it is a collision tumor consisting of an MLA alongside an AGCT, diverging from the more commonly associated epithelial ovarian tumors. Moreover, the distinct morphology of each component of the tumor is relatively identifiable in both MRI and gross photographs, which significantly enhances its diagnostic value.

In summary, we present the first case of an ovarian collision tumor with components of ACT and MLA with a unique immunophenotype and an unfavorable outcome (Figure 4). In the MRI scans, despite the challenges, variations were observed in the enhancement pattern of the T2-weighted images. This differentiation indicates the junction of distinct tumor components in a collision tumor. Identifying a collision tumor preoperatively using MRI is challenging; however, its consideration is crucial for effective diagnosis and treatment. The detection of such tumors can indicate the coexistence of both slow-growing and aggressive tumor components. This insight offers significant advantages for tailoring therapeutic interventions, underscoring the necessity of this consideration in clinical practice.

## Figures and Tables

**Figure 4:**
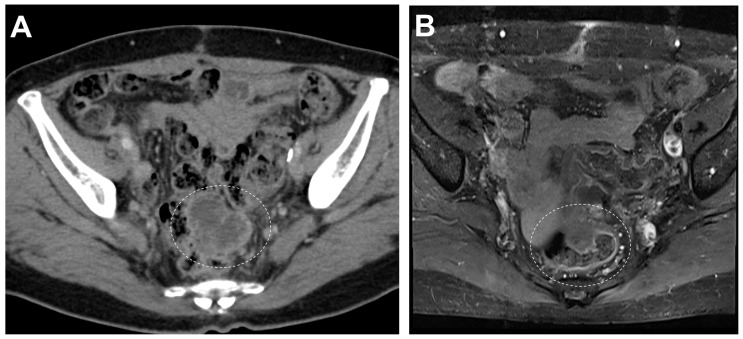
Follow-up computed tomography (**A**) taken four months after surgery revealed a lobulated cystic mass (dashed circle) measuring approximately 5 cm with irregular wall thickening within the pelvic cavity and papillary nodules around the vaginal stump. Pelvic MRI (**B**) revealed solid and cystic mass (dashed circle) and peritoneal seeding lesions up to 2 cm in diameter adjacent to the rectosigmoid junction. The treatment regimen was converted from adjuvant to palliative chemotherapy as evidence of peritoneal metastasis was found. Considering that AGCTs have been reported to have a propensity for late recurrence [29], the rapid growth of this tumor is likely attributed to a recurrence of the MLA component; furthermore, it is suggested that the collision tumor exhibits more aggressive behavior.

## Data Availability

The data presented in this study are available upon reasonable request from the corresponding author.

## Supplementary Materials

The following supporting information can be downloaded at https://www.mdpi.com/article/10.3390/diagnostics14131412/s1, Table S1: List of antibodies.
