# Supplementary material for: Collision Tumor of the Ovary: Adult Granulosa Cell Tumor and Mesonephric-like Adenocarcinoma
Source: Diagnostics (Basel). 2024 Jul 2;14(13):1412. doi: 10.3390/diagnostics14131412 (PMC11241221; doi:10.3390/diagnostics14131412)
Supplement: Supplementary file 1 [file diagnostics-14-01412-s001.zip › diagnostics-3065444-supplementary.pdf]

Supplementary Table 1. List of antibodies

Antigen	Clone	Dilution	Manufacturer
WT-1	WT49	1:1	Leica, Wetzlar, Germany
CD56a	NCAM	1:200	Leica, Wetzlar, Germany
GATA3	L50-823	1:1	Ventana, AZ, USA
TTF-1	SPT24	1:1	Leica, Wetzlar, Germany
Calretinin	CAL6	1:1	Leica, Wetzlar, Germany
CD99	12E7	1:4	Dako, Santa Clara, CA
CD10	56C6	1:4	Leica, Wetzlar, Germany
PAX-8	MRQ-50	1:1	Roche, Basel, Switzerland
Cytokeratin 7	OV-TL	1:2	Dako, Santa Clara, CA
Cytokeratin 20	Ks20.8	1:400	Dako, Santa Clara, CA
ER	6F11	1:1	Leica, Wetzlar, Germany
PR	16	1:1	Leica, Wetzlar, Germany
Vimentin	V9	1:2	Roche, Basel, Switzerland
Inhibin-alpha	R1	1:50	Cell Marque, Rocklin, CA
Napsin-A	MRQ-60	1:2	Cell Marque, Rocklin, CA
